# Prevalence and determinants of intimate partner violence among adult women in an urban community in Lagos, Southwest Nigeria

**DOI:** 10.11604/pamj.2020.36.345.24402

**Published:** 2020-08-25

**Authors:** Esther Oluwakemi Oluwole, Nneoma Claranelly Onwumelu, Ifeoma Peace Okafor

**Affiliations:** 1 Department of Community Health and Primary Care, College of Medicine, University of Lagos, Lagos, Nigeria

**Keywords:** Intimate partner violence (IPV), women, lifetime prevalence of IPV, predictors of IPV, urban community, Lagos Nigeria

## Abstract

**Introduction:**

intimate partner violence (IPV) is a global public health problem of human rights concern. It is a global issue, regardless of social, economic, religious or cultural group. Ever experienced IPV is a risk factor for many acute and chronic diseases and or stress-related conditions among women. This study determined the prevalence and predictors of lifetime IPV among women in an urban community in Lagos, Nigeria.

**Methods:**

a descriptive cross-sectional study was conducted among 400 respondents from April to September 2019. An interviewer-administered questionnaire was used to obtain information from respondents. Data analysis was done using SPSS Version 22. Descriptive analyses were performed. Associations were explored with Chi-square test; multivariate analysis was done with logistic regression at p≤5% level of significance (95% CI).

**Results:**

a total of 400 adult women aged 18-73 years who have been in an intimate relationship for at least 1 year participated in this study. The mean ± SD age of the respondents was 36.72 ± 11.74 years. Lifetime prevalence of IPV was 73.3%. The significant predictors for IPV were; being employed (OR=0.461; 95% CI=0.230-0.924); witnessed parental violence (OR=1.909; 95% CI=1.023-3.563); partner consuming alcohol (OR=1.669; 95% CI=0.999-2.788) and partner having other sexual partners (OR=2.104; 95% CI=1.174-3.771).

**Conclusion:**

community-based interventions by government and other stakeholders are needed to empower women, reduce exposure of children to IPV at home and provide enlightenment education on IPV in communities.

## Introduction

Intimate partner violence (IPV) is a global public health problem of human rights concern [1]. IPV is a major obstacle to the achievement of the sustainable development goals which aims towards gender equality and empowerment of women and girls, hence, the need for effective strategies in the control of IPV [2]. Most of the times, IPV is usually perpetrated by a husband or an intimate male partner of a woman and this is often due to the emotional attachment with or economical dependency of women on the perpetrators of violence. IPV is a global issue, regardless of social, economic, religious or cultural group [3]. Intimate partner violence refers to any behavior within an intimate relationship that causes physical, psychological or sexual harm to those in the relationship. It includes acts of physical aggression (slapping, hitting, kicking and beating), psychological abuse (intimidation, constant belittling and humiliation), forced intercourse and other forms of sexual coercion, various controlling behaviors (isolating a person from their family and friends, monitoring their movements and restricting their access to information or assistance) [3,4]. IPV affects both physical and mental health of women either directly such as injury and indirectly inform of chronic health conditions secondary to recurrent stress. Ever experienced IPV is therefore a risk factor for many acute and chronic diseases and or ‘stress-related conditions´ among women. Bruises and welts; lacerations and abrasions; abdominal or thoracic injuries; fractures and broken bones or teeth; sight and hearing damage; head injury; attempted strangulation; and back and neck injury could result from physical damage of IPV [3].

Emotional distress, suicidal thoughts or attempts were significantly higher among women who had ever experienced physical or sexual violence than those who had no experience. Also, the prevalence of injury among women who had ever experienced IPV varies from 19% in Ethiopia to 55% in Peru while, abused women were also twice more likely to report poor health, physical and mental health problems as non-abused women [4]. The civil society and governments worldwide have recognized violence against women as a public policy and human rights concern since the world conference on human rights in Vienna and the declaration on the elimination of violence against women in 1993 [4]. In Lagos State, Nigeria, to address the high burden of IPV, the government enacted a law to provide protection against victims of domestic violence in May 2007 [5]. Studies have documented between 10% and 35% of women experience domestic violence at some point in their lives [6]. A WHO multi-country study found that between 15% and 71% of the women reported physical and/or sexual violence by an intimate partner [4]. Lifetime prevalence of IPV in different European countries ranges from 10% to 36% [4]. A recent survey in 46 low/middle income countries (LMIC) reported varied levels of prevalence of psychological IPV from 6.4% in Comoros (Eastern and Southern Africa) to 34.4% in Afghanistan (South Asia), while physical and/or sexual IPV varied from 3.5% in Armenia (Europe and Central Asia) to 46% in Afghanistan [2].

Studies in sub-Saharan African and Asian on IPV reported prevalence rates ranging from 28% in Madagascar, Ethiopia (74%), India (57%) and Jordan (87%) [7]. A study in Angola reported a prevalence of 41.1% with physical (32.3%), emotional IPV (27.3%) and sexual IPV (7.4%) [8]. Another study in central region of Ghana reported a prevalence of 34%, with sexual and or physical forms (21.4%), emotional (24.6%) and economic IPV (7.4%) [9]. The situation in Nigeria is very similar to that of the entire African region, almost one in four women in Nigeria reported having ever experienced intimate partner violence [10]. A study in Lagos, southwest region, found a one-year prevalence of 29%, with majority of respondents reporting psychological (23%), physical (9%) and sexual (8%) abuse [11]. Another study in Oyo State, Nigeria found a prevalence of 31.1% [12]. Also, a study in low-income community in Southwest Nigeria, reported a lifetime prevalence of physical IPV (28.2%) [13]. Studies among pregnant women in Northern Nigeria found 28% in Zaria and 63.2% in Jos [14,15], Sokoto (30.4%) with physical violence (62.7%), psychological violence (53.5%), economic violence (48.5%) and sexual violence (57.3%) [1].

Risk factors of IPV differ in different parts of the world as domestic violence can affect women of any age, education or marital status, nationality, income, religion, age or ethnicity [6]. Generally, the factors are grouped into four; individual factors which are, young age, heavy drinking, depression, personality disorder, low academic achievement, low income, witnessing or experience violence as a child. Relationship factors includes marital conflict, marital instability, male dominance in the family, economic stress and poor family functioning. Community factors are weak sanctions against domestic violence, poverty and low social capita. Societal factors include traditional gender norms and social norms supportive of violence [3,6,9]. In Nigeria, a study reported that being young, unmarried and having a history of parental violence in the partner were significantly associated with a woman being a victim to IPV [12]. Intimate partner violence is one the most common forms of violence against women worldwide which can lead to wide array of health consequences among survivors [2]. To this end, this research aimed to assess the prevalence and determinants of intimate partner violence among adult women in an urban community in Lagos State, Nigeria.

## Methods

**Study setting:** the study was conducted in Lagos State which is located in the Southwest geo-political zone of Nigeria. The latest reports estimate the population at 21 million, making Lagos the largest city in Africa. Lagos mainland local government area (LGA) is one of the 20 LGAs in Lagos State with nine political wards and a projected population of 312,227 as at 2015 and female to male ratio of 2:1 [16]. Domestic and Sexual Violence Response Team (DSVRT) is an organization under Lagos State government, ministry of justice, committed to ensuring total eradication of sexual and gender based violence in the State. The organization provide sensitive services to victims of domestic and sexual violence while promoting healthy relationships through efforts to enhance coordinated community response to domestic and sexual violence in Lagos State and Nigeria [17].

**Study design and sampling techniques:** this was a descriptive cross-sectional study carried out between April and October 2019. Sample size was calculated using a Cochran formula (n = z^2^pq/d^2^) with a prevalence of 36.7% for IPV obtained from a previous study conducted at Ile-Ife, Nigeria [18]. An additional 10% was added to allow for missing or incompletely filled questionnaires. Hence, 400 respondents were used for the study. Only adult women (18 years and above) who were either married or in an intimate relationship for more than one year and have been resident in the community for at least 6 months were selected. Temporary visitors, business owners or those who work in the area were excluded from the study. A multi-stage sampling technique was employed in the selection of respondents. In stage one, four out of nine wards (B, E, F and I) were selected by simple random sampling through ballot. In stage two, a simple random sampling technique was used to select 10 streets in each ward from the list of streets obtained from the community development association committee. Systematic sampling method was used to select 10 houses on each street in stage three, while stage four involved the selection of household, in each house, a household was selected by simple random sampling through ballot and where more than one household met the inclusion criteria.

If a selected household had no eligible respondent, another household was selected within the same house by simple random sampling through ballot. Where no household met the criteria in a house, the next house was used. This was done until 10 households were selected per street. Stage five involved selection of participants from a household, in any household where more than one respondent was eligible to participate, a respondent was selected via simple random sampling by ballot. This was done until the required 400 respondents were recruited into the study. An interviewer administered questionnaire adapted from the WHO multi-country study on women´s health and life experiences [3] and also from studies on IPV [19,20], was used in data collection. The questionnaire was divided into four sections; sections A and B consisted of socio-demographic data of the respondent and partner respectively which were the independent variables. Section C was on the forms/types of intimate partner violence against adult women and section D was on effects/consequences of intimate partner violence which made up the dependent variables.

**Data analysis:** data was analyzed using SPSS version 22 statistical software. In assessing the experiences of different types and effects of IPV, section C and D had close ended 48 questions with options YES and NO. Lifetime prevalence of IPV was assessed based on a composite score system relating to the three domains of IPV (physical, sexual and psychological). Each positive response to any of the questions in each domain was scored 1 while any negative response had a score of 0. Assessment of the experiences of different types of IPV was done by considering any positive response to any of the questions in the different groups to have experienced that form of IPV. For controlling behavior, any positive response by the respondents to any of the 10 items under this section, were considered to have experienced controlling behavior. Frequency tables were generated and bivariate analysis was carried out with chi square. The factors found to be statistically significant were subjected to multivariate analysis to generate adjusted odds ratios at 95% confidence interval. Statistically significant level was set at p≤0.05. Ethical approval for the study was obtained from the health research and ethics committee of Lagos University Teaching Hospital (ADM/DCST/HREC/APP/3023) and permission was obtained from the authorities of Lagos Mainland local government area, Lagos State. Written informed consent was obtained from each respondent with assurance of confidentiality of information and their right to withdraw from the study at any point in time. The participants were made to understand that involvement was voluntary.

## Results

A total of 400 adult women aged 18-73 years who have been in an intimate relationship for at least 1 year participated in this study. The mean ± SD age of the respondents was 36.72 ± 11.74. More than half (57.8%) of respondents were married/cohabiting. Most (57.8%) had tertiary level of education. Majority (78%) were employed with the most (36.2%) being unskilled while only 18.9% were professionals. Higher proportion (65.2%) of the respondents have spent between 1-10 years in relationship with their spouse or partner with a mean ± SD years of 10.70 ± 11.05. About one-third (33.8%) had 1 or 2 children. Most (61.5%) of the respondent´s partners earn more and most (68.0%) of the respondents live in same house with their partners. About two-third (67.8%) of the respondents witnessed parental violence (Table 1). Respondent´s partners age ranged from 20 to 76 years with a mean ± SD age of 41.88 ± 11.55. Most (66.7%) of partners had tertiary level of education. Nearly two-third (64.3%) of the respondent´s partners consumed alcohol and most (44%) of them did so weekly. Only 23.5% of the partners used psychoactive substances, of which most (60.6%) did so daily. Over 90% of partners were employed while most (29.3%) were unskilled. Greater proportion (38.5%) of the partners had other sexual partners. Above one-tenth (13.3%) of the partners had children from other sexual partners (Table 2). The most prevalent forms of physical violence experienced by respondents were slapped (34.5%) and pushed (34.8%).

**Table 1:** socio-demographic characteristics of respondents

Variable	Frequency (n=400)	Percentage (%)
**Age group (years)**		
18-27	70	17.5
28-37	175	43.7
38-47	79	19.7
48-57	45	11.3
≥58	31	7.8
**Mean ± SD =36.72 ± 11.74**		
**Current marital status**		
Single	84	21.0
Married/Cohabiting	231	57.7
Separated/Divorced/Widowed	85	21.3
**Highest level of education attained**		
No formal education	22	5.5
Primary education	40	10.0
Secondary education	107	26.7
Tertiary education	231	57.8
**Employment status**		
Unemployed	88	22.0
Employed	312	78.0
**Occupation**	**n=312**	
Unskilled	113	36.2
Manual/non-manual skilled	67	21.5
Intermediate skilled	73	23.4
Professional	59	18.9
**Duration of relationship (years)**		
1-10	261	65.2
11-20	80	20.0
21-30	32	8.0
>30	27	6.8
**Mean ± SD=10.70 ± 11.05**		
**Number of children**		
None	119	29.7
1-2	135	33.7
3-4	103	25.8
>4	43	10.8
**Mean ± SD =1.99 ± 1.89;**		
**Earns more than partner**		
Yes	96	24.0
No	246	61.5
Don't know	58	14.5
**Lives together with partner**		
Yes	272	68.0
No	128	32.0
**Ever witnessed parental violence**		
Yes	129	32.3
No	271	67.7

**Table 2:** socio-demographic characteristics of partners

Variable	Frequency (n=400)	Percentage (%)
**Age group of partners (years)**		
20-29	36	9.0
30-39	152	38.0
40-49	127	31.7
50-59	42	10.5
≥60	43	10.8
**Mean ± SD=41.9 ± 11.6**		
**Partner's highest level of education attained**		
No formal education	28	7.0
Primary education	23	5.8
Secondary education	82	20.5
Tertiary education	267	66.7
**Alcohol use by partner**		
Yes	257	64.3
No	143	35.7
**Frequency of alcohol use**	**n=257**	
Daily	67	26.0
Weekly	113	44.0
Monthly	77	30.0
**Psychoactive substances use by partner**	**n=400**	
Yes	94	23.5
No	306	76.5
**Frequency of psychoactive substance use**	**n=94**	
Daily	57	60.6
Weekly	26	27.7
Monthly	11	11.7
**Partner's employment status**	**n=400**	
Unemployed	25	6.2
Employed	375	93.8
**Partner's occupation**	**n=375**	
Unskilled	110	29.3
Manual/non-manual skilled	96	25.7
Intermediate skilled	98	26.1
Professional	71	18.9
**Keep other sexual partners**	**n=400**	
Yes	154	38.5
No	122	30.5
Don't know	124	31.0
**Had children from other sexual partners**		
Yes	53	13.3
No	211	52.7
Don't know	136	34.0
		

**Figure 1:**
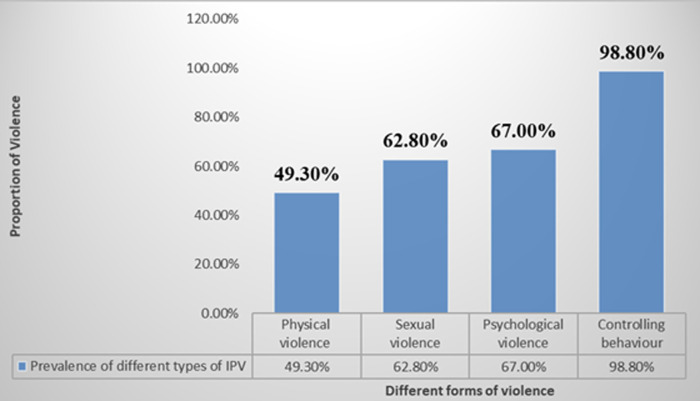
prevalence of different forms of intimate partner violence experienced by respondents

The commonest forms of sexual violence were forced to perform degrading sexual act/style (46.8%) and forceful touching of private parts (45.3%). Being insulted and called names (52.5%) were the predominant emotional/psychological violence while the least was sent packing out of the house (19.0%). The most prevalent form of controlling behavior was insisting on knowing where a partner is” (63.0%), while the least was “forced to quit job (19.2%) (Table 3). About half (49.3%) of the respondents had been exposed to at least one form of physical violence. Sexual and psychological violence had an overall prevalence of 62.8% and 67.0% respectively while controlling behavior had the highest lifetime prevalence (98.8%) (Figure 1). The overall lifetime prevalence of intimate partner violence among respondents was (73.3%). Almost three-quarter of the respondents had experienced at least one form of intimate partner violence in their lifetime. Injuries like bruises or lacerations was the commonest form of physical effects experienced by almost a quarter (23.8%) of the respondents, while the most common sexual health effects were unwanted pregnancy experienced by 24.0% of respondents and sexually transmitted infections like HIV, gonorrhea by 23.5%. The predominant psychological effect of IPV experienced by the respondents was being sad/worried (82.3%) while the least experienced were psychoactive substances abuse (4.0%) (Table 4).

**Table 3:** frequency distribution of different types of intimate partner violence

Variable	Frequency (%)	(n=400)
**Physical violence**	**Yes**	**No**
Pushed	139(34.8)	261(65.2)
Slapped	138(34.5)	262(65.5)
Choked	40(10.0)	360(90.0)
Hard blow	62(15.5)	338(84.5)
Hit/beaten	105(26.3)	295(73.7)
Thrown something at	80(20.0)	320(80.0)
Intentionally injured you with a weapon like knife, iron	31(7.8)	369(92.2)
**Sexual violence**		
Forced sexual intercourse	160(40.0)	240(60.0)
Had intercourse due to feeling of insecurities	151(37.8)	249(62.2)
Had intercourse due to threats	110(27.5)	290(72.5)
Forced to perform degrading sexual act	187(46.8)	213(53.2)
Forcefully touched your private parts	181(45.3)	219(54.7)
**Psychological/emotional violence**		
Insulted and called names	210(52.5)	190(47.5)
Embarrassed in public	149(37.3)	251(62.7)
Threatened to hurt you	105(26.3)	295(73.7)
Feels nothing you do is good enough for your partner	94(23.5)	306(76.5)
Been made afraid of partner	82(20.5)	318(79.5)
Sent packing out of the house	76(19.0)	324(81.0)
Destroyed your property	81(20.3)	319(79.7)
Partner ever walked out of the house for at least a day	89(22.3)	311(77.7)
Suspect partner of stealing your money	94(23.5)	306(76.5)
**Controlling Behaviour**		
Prevent from seeing friends	144(36.0)	256(64.0)
Restrict contact with family	83(20.8)	317(79.2)
Insist on knowing where you are	252(63.0)	148(37.0)
Neglected by partner	144(36.0)	256(64.0)
Suspects unfaithfulness	191(47.8)	209(52.2)
Complains of your busy job schedule	127(31.8)	273(68.2)
Forced to quit job	77(19.3)	323(80.7)
Seek permission to access healthcare	122(30.5)	278(69.5)
Controls spending	195(48.8)	205(51.2)
Withhold funds for basic needs	150(37.5)	250(62.5)

**Table 4:** health effects of intimate partner violence on respondents

Variable	Frequency (%)	(n=400)
**PHYSICAL EFFECTS**	**Yes**	**No**
Injuries like bruises or lacerations	95(23.8)	305(76.2)
Broken bone or loss of a tooth	40(10.0)	360(90.0)
Head injury	43(10.8)	357(89.2)
Sight or hearing impairment	58(14.5)	342(85.5)
**SEXUAL EFFECTS**		
Unwanted pregnancy	96(24.0)	304(76.0)
Abortion including unsafe abortion	71(17.8)	329(82.2)
Pregnancy complications e.g. miscarriage	43(10.8)	357(89.2)
Sexually transmitted infections	94(23.5)	306(76.5)
**PSYCHOLOGICAL/EMOTIONAL EFFECTS**		
Sad/worried	329(82.3)	71(17.7)
Feels less of yourself	181(45.3)	219(54.7)
Doubts about relationship	182(45.5)	218(54.5)
Suicidal thoughts	54(13.5)	346(86.5)
Suicidal attempts	21(5.3)	379(94.7)
Alcohol abuse	86(21.5)	314(78.5)
psychoactive substances abuse	16(4.0)	384(96.0)
Admission in a hospital	58(14.5)	342(85.5)
Loss of job	50(12.5)	350(87.5)

With bivariate analysis, higher proportions of those who were separated/divorced /widowed (87%, p<0.05); primary or no formal education (85% and 82%, p=0.05); unemployed (84%, p<0.05); engaged in skilled occupation (79%, p<0.05); or witness of parental violence (86%, p<0.001) had experienced IPV. The main partner characteristics significantly associated with IPV perpetration include level of education as those with less than tertiary education are more prone to be a perpetrator of IPV (82%, 84%, 87%, p<0.05); with skilled occupation (83.3%, p<0.001); alcohol consumption (78%, p=0.003), daily intake of alcohol (91%, p<0.001), psychoactive substance use (85%, p=0.003), and had other sexual partners (79%, p=0.004). Binary logistic regression shows that respondent´s employment status, witnessed of parental violence, alcohol consumption by partner and partner who had other sexual partners were independent predictors of lifetime experience of IPV. Women who were employed (OR=0.461; 95% CI=0.230-0.924); were less likely than those who were not employed to report ever being abused. Respondents who had witnessed parental violence (OR=1.909; 95% CI=1.023-3.563); whose partner consumed alcohol (OR=1.669; 95% CI=0.999-2.788) and partners who had other sexual partner(s) (OR=2.104; 95% CI=1.174-3.771) were all two times more likely to experience IPV (Table 5).

**Table 5:** predictors of lifetime experience of IPV among respondents

Variables	Odd ratio	95% CI	p-value
**Current marital status**			
Single	1		
Married	0.903	0.486-1.678	0.747
Separated/Divorced/Widowed	2.179	0.915-5.189	0.079
**Level of Education**			
No formal	1		
Primary	1.735	0.365-8.258	0.489
Secondary	0.992	0.258-3.819	0.990
Tertiary	0.984	0.256-3.782	0.982
**Respondents’ employment status**			
Unemployed	1		
Employed	0.461	0.230-0.924	0.029
**Respondents witness of parental violence**			
No	1		
Yes	1.909	1.023-3.563	0.042
**Alcohol consumption by partner**			
No	1		
Yes	1.669	1.000-2.788	0.050
**Psychoactive substances use by partner**			
No	1		
Yes	1.336	0.671-2.661	0.410
**Other sexual partners**			
No	1		
Yes	2.104	1.174-3.771	0.012

## Discussion

Violence against women by an intimate partner contributes significantly to the ill-health of women worldwide and it is both a consequence and a cause of gender inequality [3,21]. Despite the imperative nature of the problem, there is lack of adequate data or information on IPV, due to cultural acceptability of intimate partner violence leading to underreporting and fear of disclosure [22]. The sociodemographic characteristics of respondents in this study are comparable to those of similar studies in Lagos and Benin, Nigeria [19,23]. The similarity could be attributed to the fact that the studies were all conducted in cosmopolitan urban areas. In this study, almost all (98.8%) respondents have ever had the experience of controlling behavior of the partners. This finding is similar to the report of a study involving the use of secondary data from NDHS 2008 and other studies in Kano, Lagos and Sokoto Nigeria [15,19,24,25]. The WHO study findings has suggested that the experience of physical or sexual violence, or both, tends to be accompanied by highly controlling behavior by intimate partners [3]. Controlling behavior reflects the increased vulnerability of women to be violated and this shows the patriarchal dominance of male in the family and the social norms that encourage men to exercise control over women. Controlling behavior has been documented to be a precursor of violence and its directly related with increased likelihood of acts of violence [15,19]. The results of our study revealed a high prevalence of psychological IPV (67%).

This prevalence is slightly lower than the prevalence of 85% reported in a previous study in Lagos [19], probably due to the fact that controlling behavior was excluded in the estimation of the overall prevalence in our study. However, the figure is higher compared to the study in Sokoto (53.5%) [15], Lagos (23%) [10] and Ibadan (50.1%) [12]. This may be due to difference in study population as our study involved all women and not among pregnant women only. This finding also, points to increasing prevalence of IPV. Most (62.3%) of the respondents had experienced sexual form of IPV in their lifetime in this study. Contrary to other reports in Lagos (33.8%) [19], Ibadan (13.6%) [12], Lagos (8%) [10], Sokoto (57.3%) [15] and Benin (18.7%) [23]. This could be due to high and daily alcohol consumption among the majority (64.3%) of the respondents´ partners which is found to be statistically significant with IPV in this study. Similar finding were reported by a study in Southwest Nigeria [12]. We found a prevalence of 49.3% for physical violence in this study and this falls within the reported global range of 15% to 71% [3]. However, lower to report of studies in Lagos (50.5%), [19] South-south Nigeria (60.2%), [26] and Sokoto (62.7%) [15]. But higher compared to studies in Southwest Nigeria (28.2%) [12], Edo State, Nigeria (16.9%) [23] and central region of Ghana (32.2%) [8]. This might be explained by differences in the cultural norms of the study populations and time differences. The overall lifetime prevalence of IPV in this study was 73%.

This figure is comparable to that in previous studies conducted in Ile-Ife (77.3%) [27] and Benin, Nigeria (76.0%) [26]. However, higher compared to studies in Edo State, Nigeria (32%) [23], Sokoto, Nigeria (33%) [15], Ibadan, Nigeria (59.5%) [12] and the study that used data from the population-based 2013 Nigerian Demographic and Heath Survey (DHS) reported 23.5% [9]. Report by authors across different regions in Nigeria have reported prevalence of IPV ranging from 42% in the North [25], 29% in the Southwest [9], 78.8% Southeast [28], to 41% in the South-south [29]. Furthermore, studies in Western Turkey (39.0%) [3] and Japan (15%) [30]. The range of lifetime prevalence of physical or sexual violence, or both by an intimate partner has been documented to range from 15% to 71%. The wide disparities in prevalence across different regions has been documented by WHO report as due to the different social and macro-structural context in the different regions [3]. The physical damage resulting from IPV can include: bruises and welts; lacerations and abrasions; abdominal or thoracic injuries; fractures and broken bones or teeth; sight and hearing damage; head injury; attempted strangulation; and back and neck injury [3]. In this study, psychological health effects were the most frequently experienced health effect by the respondents. Among the physical effect of IPV experienced by respondents were injuries while the most common sexual IPV health effects were unwanted pregnancies, sexually transmitted infections and pregnancy complications.

Similar findings have been reported by a study in Northwest Ethiopia [31]. Research evidence has documented the profound impact of a violent partner on a woman´s health. IPV has been linked to a host of different health outcomes, both immediate and long-term. IPV leads to injuries, ranging from cuts and bruises to permanent disability and death [1]. A study in Canada reported that 43% and 50% of women injured during IPV respectively received medical care and had to take time off from work [32]. WHO multi-country study found the prevalence of injury among women who had ever been physically abused by their partner ranged from 19% in Ethiopia to 55% in Peru. Abused women were also twice as likely as non-abused women to report poor health and physical and mental health problems, even if the violence occurred years before [3]. Regarding factors associated with IPV in this study; respondents marital status, level of education, employment status, type of occupation, or witnessed of parental violence and partner´s level of education, partner´s type of occupation, partner´s alcohol consumption and psychoactive substance use by partners have also been reported by authors in other studies in Sokoto [15], Edo State, Nigeria [23,26]. Also in Ghana [8] and Angola [7]. IPV is believed to be the outcome of a dynamic interaction of risk and protective factors that range from broad social factors to individual risk factors” [3].

However, further analysis showed that respondents employment status, witness of parental violence, partner´s alcohol consumption and partner having other sexual partners were the independent predictors of lifetime experience of IPV in our study. Similar findings have been documented by studies [7,13,15,19,23]. This finding buttresses the fact that the determinants of IPV varies from place to place and settings to settings and is an interaction of different factors which include individuals, relationships, community and societal factors. This study was community based with sound and well detailed methodology, strict adherence to ethical rules in data collection followed by detail analysis and interpretation of the data. In addition, due to the sensitive nature of the topic, the face-to-face method of interview could have led to under-reporting but to minimize this, interviews were conducted privately. If partner was around, topic was disguised at the beginning and not within the hearing of partner or re-scheduled to a more convenient time. These are the strength of our study. However, the cross-sectional nature of the study does not allow for causal inferences. Furthermore, data was collected from only one local government areas in Lagos State, though representative of an urban LGA but generalization to the entire State cannot be made. However, this study adds to the body of evidence on IPV and may be applicable to other similar urban similar communities.

## Conclusion

The prevalence of IPV in this study was high. Controlling behaviors of respondent´s partners had a very high prevalence, replicating the fact that the respondents lives in a patriarchal society with masculine domination. Psychological violence was the most prevalent form of IPV in this study. Several individual and spousal/partner´s characteristics predict lifetime experience of IPV. Community based interventions by government and other stakeholders are needed to empower women, reduce exposure of children to IPV at home, provide enlightenment education on IPV in communities. Also, strategies of reducing alcohol consumption among the male partners will also go a long way in reducing the prevalence of IPV.

### What is known about this topic

- Intimate partner violence (IPV) is an important public health issue and human rights concern;
- Intimate partner violence affects both physical and mental health of women either directly such as injury and indirectly inform of chronic health conditions secondary to recurrent stress.

### What this study adds

- The present study adds to the current knowledge by providing a lifetime prevalence of different forms of IPV among women in an urban setting in Southwest Nigeria;
- The study provides further understanding of factors and determinants of intimate partner violence and thus help policy makers and stakeholders to device strategies for control programmes in the community.
